# DNA analysis of a 30,000-year-old *Urocitellus glacialis* from northeastern Siberia reveals phylogenetic relationships between ancient and present-day arctic ground squirrels

**DOI:** 10.1038/srep42639

**Published:** 2017-02-16

**Authors:** Marina Faerman, Gila Kahila Bar-Gal, Elisabetta Boaretto, Gennady G. Boeskorov, Nikolai E. Dokuchaev, Oleg A. Ermakov, Fedor N. Golenishchev, Stanislav V. Gubin, Eugenia Mintz, Evgeniy Simonov, Vadim L. Surin, Sergei V. Titov, Oksana G. Zanina, Nikolai A. Formozov

**Affiliations:** 1 Laboratory of Bioanthropology and Ancient DNA, Faculty of Dental Medicine, The Hebrew University of Jerusalem, Jerusalem 91120, Israel; 2 Koret School of Veterinary Medicine, The Robert H. Smith Faculty of Agriculture, Food & Environment, The Hebrew University of Jerusalem, Rehovot 76100, Israel; 3 D-REAMS Radiocarbon Laboratory, Scientific Archaeology Unit, Weizmann Institute of Science, Rehovot 76100, Israel; 4 Diamond and Precious Metals Geology Institute of the Siberian Branch of the Russian Academy of Sciences, Yakutsk 677007, Russian Federation; 5 Institute of Biological Problems of the North, Far-East Branch of the Russian Academy of Sciences, Magadan 685000, Russian Federation; 6 Department of Zoology and Ecology, Penza State University, Penza 440026, Russian Federation; 7 Laboratory of Theriology, Zoological Institute, Russian Academy of Sciences, Saint Petersburg 199034, Russian Federation; 8 Soil Cryology Laboratory, Institute of Physicochemical and Biological Problems in Soil Science, Russian Academy of Sciences, Pushchino 142290, Russian Federation; 9 Papanin Institute for Biology of Inland Water, Russian Academy of Sciences, Borok 152742, Russian Federation; 10 Institute of Systematics and Ecology of Animals, Siberian Branch of Russian Academy of Sciences, Novosibirsk 630091, Russian Federation; 11 Tomsk State University, Tomsk 634050, Russian Federation; 12 National Research Center for Hematology, Russian Ministry of Health, Moscow 125167, Russian Federation; 13 Department of Vertebral Zoology, Faculty of Biology, Lomonosov Moscow State University, Moscow 119991, Russian Federation

## Abstract

In contrast to the abundant fossil record of arctic ground squirrels, *Urocitellus parryii*, from eastern Beringia, only a limited number of fossils is known from its western part. In 1946, unnamed GULAG prisoners discovered a nest with three mummified carcasses of arctic ground squirrels in the permafrost sediments of the El’ga river, Yakutia, Russia, that were later attributed to a new species, *Citellus (Urocitellus) glacialis* Vinogr. To verify this assignment and to explore phylogenetic relationships between ancient and present-day arctic ground squirrels, we performed ^14^C dating and ancient DNA analyses of one of the El’ga mummies and four contemporaneous fossils from Duvanny Yar, northeastern Yakutia. Phylogenetic reconstructions, based on complete *cytochrome b* gene sequences of five Late Pleistocene arctic ground squirrels and those of modern *U. parryii* from 21 locations across western Beringia, provided no support for earlier proposals that ancient arctic ground squirrels from Siberia constitute a distinct species. In fact, we observed genetic continuity of the *glacialis* mitochondrial DNA lineage in modern *U. parryii* of the Kamchatka peninsula. When viewed in a broader geographic perspective, our findings provide new insights into the genetic history of *U. parryii* in Late Pleistocene Beringia.

Readers of “The Gulag Archipelago” by Aleksandr I. Solzhenitsyn might remember how the book starts: “In 1949 some friends and I came upon a noteworthy news item in *Nature*, a magazine of the Academy of Sciences. It reported in tiny type that in the course of excavations on the Kolyma River a subterranean ice lens had been discovered which was actually a frozen stream - and in it were found frozen specimens of prehistoric fauna some tens of thousands of years old” (p. *ix*)^1^. That very same news item in *Nature (‘Priroda’*) continued by reporting what Solzhenitsyn did not: that in May 1946 unnamed prisoners of GULAG recovered a nest with three complete mummified carcasses of arctic ground squirrels at a depth of 12.5 meters of the permafrost sediments of the El’ga river (the upper Indigirka river basin, Yakutia)^2^,^3^. The carcasses were extremely well preserved and “smelled of dampness immediately upon their recovery but lost the smell after having air-dried and remained in a stable condition resembling that of the mummies” (p. 76)^3^ (Fig. 1). It was suggested that they had lain in the permafrost for at least 10–12 thousand years^3^.

Two of the carcasses were first examined by B.S. Vinogradov who assigned them to a new species, *Citellus (Urocitellus) glacialis* Vinogr., based on a number of distinct morphological features which discriminated these ancient arctic ground squirrels from those of the present-day northeastern Siberia^4^. It is noteworthy that B.S. Vinogradov himself as well as others later^5^ questioned this assignment because of certain similarities in size and morphology of the El’ga specimens to some North American, especially old-aged, arctic ground squirrels. Following a recent generic revision of the ground squirrel genus *Spermophilus*^6^, we refer here to arctic ground squirrels as *Urocitellus parryii*.

Mitochondrial DNA-based (mtDNA) studies on modern arctic ground squirrels support a scenario according to which all major divergence events in the Late Pleistocene occurred in North America^7^,^8^. A detailed examination of mtDNA variation in arctic ground squirrels throughout Alaska and the adjoining Yukon Territory revealed four non-overlapping geographic clades–Arctic, Beringia, Southeastern and Southwestern, possibly dated to the Middle Pleistocene^9^. This and two later studies^10^,^11^ indicated that southwestern Alaska populations, found south and west of the Alaska Range, were strongly differentiated from all other arctic ground squirrels of northwestern North America. Most recently, a study of a large mtDNA dataset of the genus *Urocitellus*, including taxon sampling at the subspecies level for *U. parryii*, demonstrated that there are two distinct *U. parryii* mtDNA clades (“Northern Beringia” and “Southern Beringia”) that currently have amphi-Beringian distribution^12^. It is noteworthy that two samples of *U. p. stejnegeri* from the Kamchatka peninsula included in that study were placed within the Southwestern clade. The authors suggested that multiple colonization events had occurred in the history of the genus; however, their number and timing remained uncertain.

It has been shown that for an accurate reconstruction of population history both modern and ancient DNA (aDNA) data are required^13^. Combining genetic findings with direct radiocarbon dating of fossils significantly improves our understanding of population dynamics over time. This comprehensive approach has been used to examine climate and anthropogenic effects on the demographic history of large-bodied mammals during the Late Quaternary period, revealing that different species respond differently to these effects^14^. Similarly, collared lemming and the narrow-skulled vole, two key prey rodents of the Arctic ecosystem, have been shown to respond very differently to climate change^15^. Still, the majority of these studies have focused on large- and medium-size mammals (steppe bison^16^, cave bear^17^, woolly mammoth^18^, wild horse^19^, cave lion^20^, wolf^21^ etc.) while small mammals remain underrepresented.

With the aim of verifying the previous assignment of *U. glacialis* as a distinct species and to explore phylogenetic relationships between ancient and modern arctic ground squirrels, we performed direct ^14^C dating and assessed mtDNA (*cytochrome b* gene) variation in ancient arctic ground squirrels from northeastern Siberia in comparison to that in modern *U. parryii*. There is abundant and long-standing fossil evidence of arctic ground squirrels in northwestern North America^22^, with much of the prehistoric range associated with the mammoth-steppe ecosystem^23^,^24^, only few fossils are known from northeastern Asia^25^.

This study included five arctic ground squirrels originating from two permafrost sites in northeastern Siberia: the El’ga River, a left tributary of the upper Indigirka river (N = 1), and the Duvanny Yar (N = 4) located on the right bank of the lower Kolyma river, Yakutia, and separated from the former by ~1000 km. In addition, museum specimens of modern *U. parryii* were selected from 21 locations in northeastern Siberia and the Kamchatka peninsula in order to obtain a geographically representative sample across their present-day habitat in western Beringia (Fig. 2 and Supplementary Table S1). We designated our samples as Gla (ancient), BerR (Beringia, Russia) and Kam (the Kamchatka peninsula).

## Results and Discussion

### Radiocarbon dating

A direct ^14^C age of 29,450 ± 925 uncal. years BP was determined by accelerator mass spectrometry (AMS) on a liver sample of *U. glacialis* (Gla1, RTK 6386) (Fig. 3 and Supplementary Information). The ±1σ and ±2σ calibrated ranges were estimated at 33,990-31,990 (68.2% probability) and 34,920-31,250 (95.4% probability) years cal BP, respectively. Radiocarbon dates from the Duvanny Yar, 31,800 ± 310 uncal. years BP, were available previously based on the contents of rodent burrows^26^.

### Tracing the *glacialis* mtDNA lineage in present-day *U. parryii*

We retrieved and analyzed complete *cytochrome b* gene sequences (*cyt b*, 1140 bp) for each of the five ancient arctic ground squirrels. DNA of *U. glacialis* was extracted from 3–5 mg of tissue (bone, skin, liver) using a slightly modified silica-based procedure^27^,^28^ (see, Methods section for details). The sequences obtained for *U. glacialis* (Gla1) were replicated independently by two laboratories to exclude ancient DNA degradation as a possible cause for the polymorphic nucleotide positions observed. DNA extraction from four fossil arctic ground squirrels from the Duvanny Yar was carried out from 10–20 mg of bone powder using a phenol/chloroform protocol after overnight pretreatment with proteinase K at 37 °C. Eighteen short, spanning 96–140 bp, overlapping sequences of mitochondrial *cyt b* gene were targeted by PCR using newly designed primers based on the sequence of modern *U. parryii* from Atka, Magadanskaya oblast (GenBank accession number AF157896) (Supplementary Table S2). DNA from museum dry and ethanol-preserved specimens was extracted using a standard phenol/chloroform protocol and amplified either in four (312–421 bp, museum dry specimens) or in two (866 and 908 bp, ethanol-preserved samples) overlapping fragments using newly designed primers (Supplementary Table S2). Each set of experiments was accompanied by appropriate blank controls.

The Gla1 sequence and those of the Duvanny Yar samples (Gla2a,b,c and d) were very similar to each other but differed significantly from those of modern arctic ground squirrels from 17 geographic localities across northeastern Siberia generated in this study. However, they shared 28 unique nucleotide positions, defining the *glacialis* mtDNA lineage, with those from the Kamchatka peninsula (Supplementary Table 3). The rest of the specimens of modern *U. parryii* examined in this study were found to belong to the Beringia clade, previously reported from northwestern North America^9^,^11^. We then expanded our analysis to include published data on populations from Alaska and Canada and used *U. richardsonii* and *U. columbianus* as outgroups (see, Methods section for GenBank accession numbers of the sequences included). For follow-up, we accepted previously used geographic clade designation, namely, Arctic (Arc), Beringia (Ber), Southwest (SW), Southeast (SE)^11^. It is noteworthy that 20 of 28 unique nucleotide positions, characteristic of the *glacialis* mtDNA lineage, were observed in the SW clade from northwestern North America.

We estimated that 71.89% of the total genetic variance resulted from differences between the groups and 28.11% within the groups (P < 0.001). Haplotype diversity (*h*) was high both in the ancient and modern samples from northeastern Siberia and the Kamchatka peninsula (ranging from 0.964 ± 0.023 to 1.000 ± 0.052)^29^. The mean interspecific *p*-distances between Late Pleistocene arctic ground squirrels and those inhabiting northeastern Siberia at present ranged from 1.3 ± 0.3% in Gla-Kam to 3.4 ± 0.5% in Gla-Ber pairs. The western (Asia) and eastern (North America) *U. parryii* of the Ber clade showed the lowest mean pairwise *p*-distance of 0.9 ± 0.2%, reflecting their close genetic relationship and recent divergence following the final disappearance of the Bering Land Bridge.

**Figure 2:**
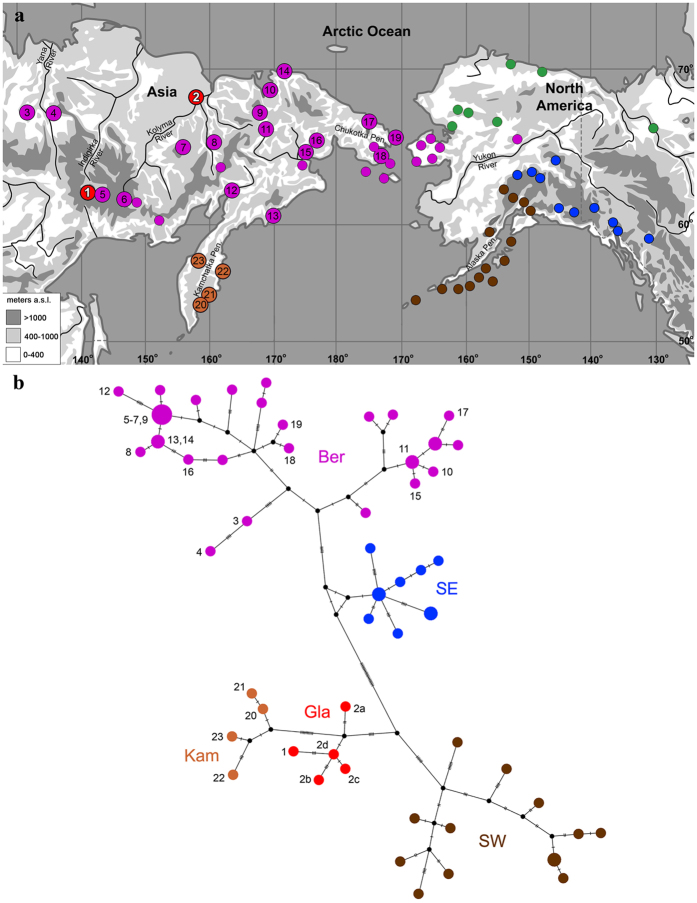
(**a**) Map of sampling localities; (**b**) MJ network of 55 *cyt b* haplotypes in subarctic *U. parryii*. Specimens examined in this study are numbered from 1 to 23 and colored in red (Gla), terra cotta (Kam) and purple (BerR). Locality numbers cross-reference Supplementary Table S1. Specimens from previous studies^9^,^11^ are depicted in purple (Ber), brown (SW), blue (SE) and green (Arc). The map was created using Inkscape 0.48.4 software (https://inkscape.org). The network was created using PopART v.1.7 software (http://popart.otago.ac.nz). The areas of the circles are proportional to the haplotype frequencies. Number of hatch marks corresponds to the number of mutational steps. Black circles depict haplotypes not observed in the sample.

To further examine the phylogenetic relationships between the sampled locations we constructed a median-joining network of the observed *cyt b* haplotypes using PopART v.1.7 software^30^. Figure 2b displays the MJ network of the 55 haplotypes representing 65 subarctic *U. parryii*. The MJ network revealed four distinct clusters: Gla+Kam, SW, SE and Ber+BerR. According to the MJ genealogy Late Pleistocene arctic ground squirrels are ancestral to those currently inhabiting the Kamchatka peninsula. The Ber+BerR group is represented by two sub-lineages each including western and eastern Beringia samples intermingled with one another. Two sub-lineages seen in the SW cluster represent mainland Alaska and adjoining islands, respectively.

We observed the same pattern of genetic affinities in a maximum likelihood (ML) tree constructed from 27 newly obtained complete *cyt b* gene sequences and 45 previously published ones (Fig. 4). Two major clades were present within subarctic *U. parryii*: Gla/Kam/SW and Ber/SE, each containing two well-supported subclades. The ancient specimens branched together with modern *U. parryii* from the Kamchatka peninsula, and next to those from the Alaska Peninsula, but not with *U. parryii* inhabiting northeastern Siberia at present.

### Defining the diversification timeline for arctic ground squirrels in the Late Pleistocene

We applied a Bayesian phylogeographic approach to infer the timing of dispersals for ancient arctic ground squirrels in eastern and western Beringia (Fig. 5). The original dataset was examined using TipDatingBeast^31^ and found to be rate informative for tip-dating analysis using BEAST (Supplementary Fig. S1). For tip calibration, the radiocarbon ages of the ancient specimens were used: a mean of 33,075 years BP for *U. glacialis* and 31,800 years BP for the specimens from the Duvanny Yar^26^. Mean and 95% HPD values of TMRCA of the nodes are given in Supplementary Table S4.

The results of tip-calibrated BEAST analysis allowed us to suggest a plausible scenario for the diversification of subarctic ground squirrels in Late Pleistocene Beringia and to provide a defined timetable for these events. We estimated that 123 kyr ago, during the Kazantsevo interglacial, roughly corresponding to the Pelukian transgression (marine isotopic stage 5e (MIS 5e), 125–115 kyr BP)^32^, subarctic *U. parryii* had become separated from the Arc/*U. richardsoni* group, that remained to the north of the Brooks Range. The subarctic *U. parryii* populations had shared a most recent common ancestor (MRCA) 104 kyr ago and, as hinted by our findings, since then some of them might have crossed the Bering Land Bridge westward to northeastern Siberia. Following the Simpsonian transgression (88–70 kyr BP)^32^ they became isolated from the ancestral pool in eastern Beringia. As a result, the ancestors of the *Glacialis* family and of modern *U. parryii* from the Kamchatka and the Alaska peninsulas, with the MRCA dated to 65 kyr BP, spread further into northeastern Siberia as far as the upper Indigirka river in the west and the lower Kolyma river in the north, while the ancestors of arctic ground squirrels, currently found on both sides of the Bering Strait and in southeastern Alaska (Ber and SE clades), remained on the North American continent.

The Karginsky/Middle Wisconsinan interglacial (55–25 kyr BP, MIS 3 [ref. 32]), which included two cooling (ca. 40–45 and 30–35 kyr BP) and three warming events^33^ caused a further fragmentation of the natural habitat of arctic ground squirrels. Following the beginning of the Last Glacial Maximum (LGM), the SW population established itself in the Alaska peninsula and adjacent islands, shortly after *U. glacialis* of Yakutia had disappeared. The *Glacialis* family survived the extinction in one of the LGM refugia in western Beringia, the Kamchatka peninsula. These findings provide no support for earlier proposals that ancient arctic ground squirrels from northeastern Siberia constituted a distinct species. On the contrary, the observed genetic continuity of the *glacialis* mtDNA lineage in modern *U. parryii* allows us to refer to it as a subspecies, *U. parryii glacialis*.

Regarding eastern Beringia, our data were compatible with an Alaskan center of origin for the Ber/SE group, with an estimated time of the MRCA of 44 kyr BP, and showed that the Ber/BerR group had expanded its habitat into northeastern Siberia 29 kyr ago, corresponding in time to the onset of the LGM sea-level lowstand (26–19 kyr BP)^34^. Although two sub-clades within the Ber/BerR group were identified in this study, dated to 25 and 20 kyr BP, respectively, each of them included individuals from both western and eastern Beringia, reflecting continuous gene flow during the Sartan/Late Wisconsinan glacial (MIS 2, 25–15 kyr BP)^32^, which was interrupted by Holocene sea-level rise reopening the Bering Strait.

While recognizing the limitations of a single-gene approach one should note that (1) close genetic affinities between the Gla/Kam and SW clades identified here are in agreement with the morphological resemblance of *U. glacialis* and some of North American *U. parryii* recorded previously^4^,^5^; (2) modern mtDNA-based studies of *U. parryii* in northwest North America have highlighted the distinctiveness of southwestern Alaska populations^9^,^10^,^11^,^12^; and (3) major geographic patterns of mitochondrial and nuclear DNA structure have been reported to be concordant for *U. parryii* of eastern Beringia, with the exception of the relationship between the Ber and SW groups^11^. Our findings allow us to resolve the latter discrepancy by providing ‘ice-cold’ evidence of close genetic affinities between the *Glacialis* family from Yakutia and southwestern Alaska populations and to suggest a possible Siberian origin of the latter followed by an eastward expansion.

Previous DNA-based studies argued that the genetic structure of modern arctic ground squirrels resulted from *in-situ (i.e*. eastern Beringia) diversification in the Late Pleistocene^11^,^12^. These studies, largely limited to northwestern North America samples, have shown that two of the four major mtDNA lineages identified so far (Ber and SW) are present on both sides of the Bering Strait^12^. Using tip-calibrated BEAST analysis we demonstrate that diversification of arctic ground squirrels falls well within the Late Pleistocene period, as suggested previously, and provide a defined timeline for these events. For the first time we reveal an early divergence of the Gla/Kam/SW and Ber/SE groups of subarctic *U. parryii* going back to 104 kyr BP. We further show that the Ber clade, having originated in Alaska, is now widely spread all over western Beringia, except for the Kamchatka peninsula. We describe a new mtDNA lineage (Gla-Kam) in arctic ground squirrels of western Beringia. This lineage appears to be a sister-clade to that of modern subarctic ground squirrels from southwestern Alaska and shares with the latter an MRCA that lived 65 thousand years ago. Although the Gla-Kam-SW group might have evolved in eastern Beringia, the presence of the *glacialis* mtDNA lineage 30 thousand years ago in northeastern Siberia is supported by direct radiocarbon dating and suggests that the onset of colonization of western Beringia by subarctic *U. parryii* from northwestern North America was earlier than previously thought.

In order to verify this hypothesis, further DNA studies based on a larger number of fossils from western and eastern Beringia, sampled if available at critical time-points as determined here, would be needed.

## Methods

### Specimens

Five ancient arctic ground squirrels from two permafrost sites in northeastern Siberia, Russian Federation, were examined: the El’ga river, a left tributary of the upper Indigirka river (ZIN-34046) and the Duvanny Yar located on the right bank of the lower Kolyma river. Three fossil arctic squirrels from the Duvanny Yar (IGDPM-6391, P-1311 and P-1322) were recovered from burrows^25^,^26^ while specimen P-Up4 was not found *in situ* but collected from exposed sediments on the bank of the lower Kolyma river, in the upper region of the Duvanny Yar. Data on morphology and morphometrics of the specimens and on associated palaeoecological findings are available elsewhere^4^,^25^,^26^. Museum specimens of modern *U. parryii* were sampled from 21 locations across northeastern Siberia and the Kamchatka peninsula from two sources: dry (bone and teeth, N = 19) and ethanol-preserved specimens (liver, N = 2) (Fig. 2 and Supplementary Table S1).

### Radiocarbon dating

**Figure 3:**
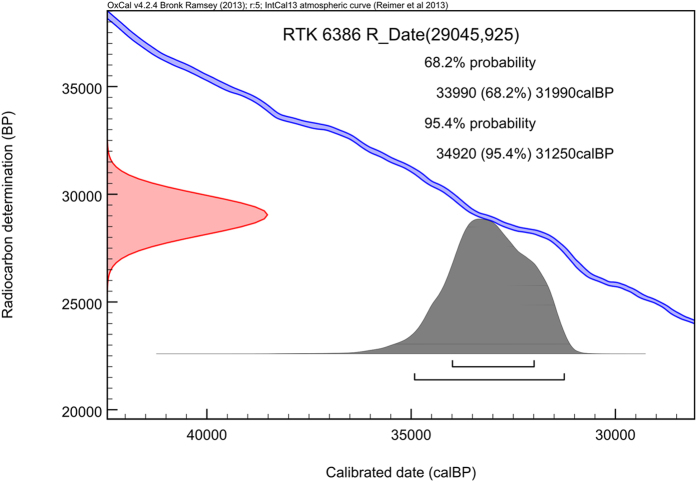
Radiocarbon dates and probability distribution of the calibrated ranges.

**Figure 4:**
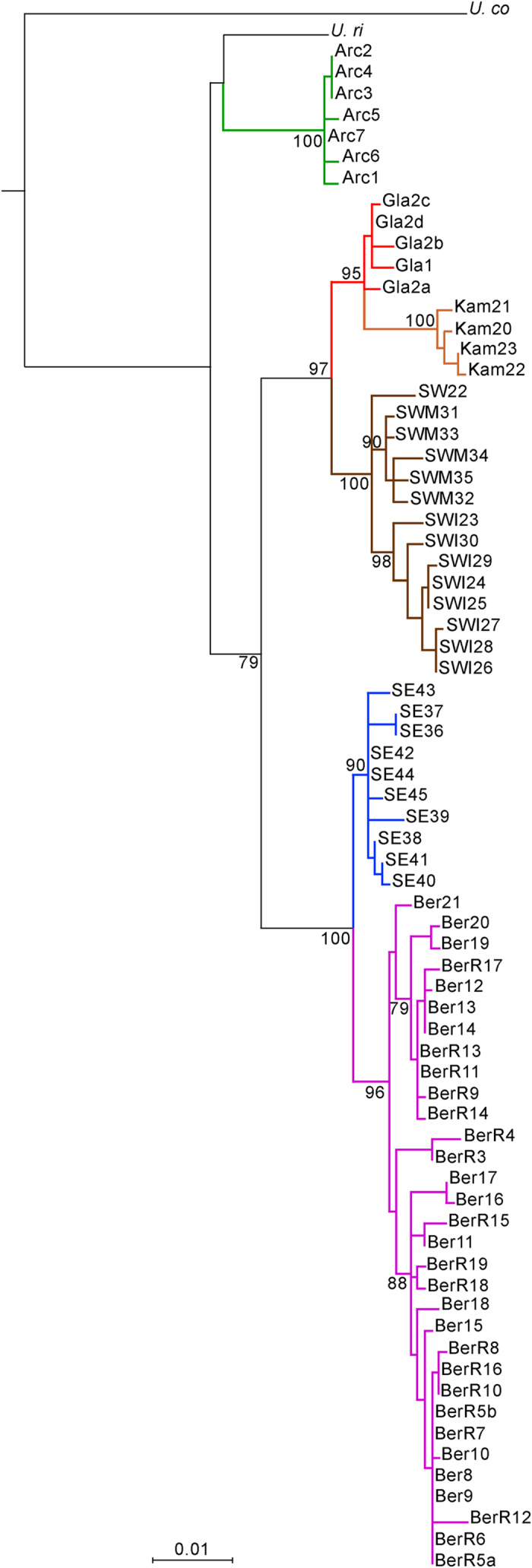
Maximum likelihood tree based on 72 complete *cytochrome b* gene sequences (1140 bp) of arctic ground squirrels. Bootstrap values are shown next to the node. *U. columbianus* and *U. richardsonii* were used as outgroups. Names and colors cross-reference Supplementary Table S1 and Fig. 2, respectively.

**Figure 5:**
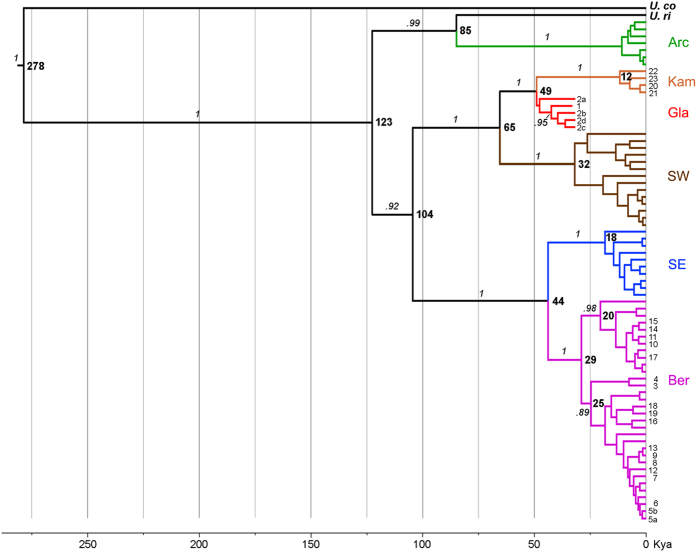
A time-calibrated maximum clade credibility mitochondrial genealogy obtained from a Bayesian analysis of complete *cytochrome b* gene sequences in 72 arctic ground squirrels. Main node values are indicated in kyr BP. Bayesian posterior probabilities are given in italic above the line. Names and colors cross-reference Supplementary Table S1 and Fig. 2, respectively.

After pre-treatment^35^, collagen was extracted from a liver sample of *U. glacialis* and analyzed using Fourier Transform Infrared (FTIR) spectrometry (MIDAC Corporation, Costa Mesa, CA, USA). ^14^C determination was performed by accelerator mass spectrometry (AMS). Radiocarbon dates were reported in conventional ^14^C years BP, corrected for isotopic fractionation based on the stable carbon isotope ratio (δ^13^C value). Calibrated ages in calendar years were obtained using the IntCal13 terrestrial calibration model^36^ by means of OxCal v. 4.2.4 [refs 37 and 38] (Fig. 3 and Supplementary Information).

### DNA extraction and amplification

Laboratories 1 and 2: Three samples of *U. glacialis* (ZIN-34046) - bone, skin and liver, were available for DNA analysis. The analyses were performed independently by 2 laboratories in facilities strictly dedicated to ancient DNA (aDNA) research (Jerusalem and Rehovot) and physically separated from the post-PCR and modern DNA working area. Moreover, this was the first time that, the Jerusalem laboratory has handled any animal specimens. Since the tissue samples had been transported in 70% ethanol, they were air-dried at room temperature and a sub-sample was removed and ground to a fine powder. DNA was extracted using a modified silica-based method^27^,^28^ as follows. Two to five milligrams of each sample were collected into a 1.5-ml Eppendorf tube containing 500 μl GuSCN-solution (4 M GuSCN; 0.1 M Tris-HCl, pH 6.4; 0.002 M EDTA, pH 8.0; 1.3% Triton) and incubated with gentle agitation at 56 °C overnight. DNA was isolated by binding to 10 μl of an in-house made silica suspension in the presence of 1 ml 6 M NaI on ice for an hour, washed twice with 0.5 ml of ice-cold 70% ethanol, air-dried for an hour and then eluted in 100 μl of sterile PCR water at 56 °C for an hour. DNA of *U. glacialis* was amplified using 18 sets of primers spanning short amplicons of 96–140 bp (Supplementary Table S2), which were designed based on the complete *cytochtome b* sequence of a modern *U. parryii* from Atka, Magadanskaya oblast (GenBank accession number AF157896). Two to five microliters of the final volume of each aDNA extract were subjected to hot-start PCR amplification in a 25 μl reaction containing 1 × buffer, 10 pmol of each primer, 0.2 mM of each dNTP, 1.5 mM MgCl_2_ and 1 unit of AmpliTaq Gold DNA polymerase (Applied Biosystems, USA). PCR was performed for 45 cycles as follows: 94 °C–30 sec, 50 °C–30 sec, 72 °C–30 sec. In order to remove the inhibitors and to concentrate aDNA extracts bovine serum albumin (BSA) was added to each tube at a final concentration of 0.8 mg/ml and/or Microcon YM-100 (Millipore Corporation, MA, USA) were applied following the manufacturer’s guidelines. PCR products were verified on 2% agarose gels stained with ethidium bromide. Prior to sequencing 5 microliters of the PCR products were cleaned using Exonuclease Shrimp Alkaline Phosphatase (Exo-Sap IT, Pharmacia) following the instructions of the manufacturer. Sequencing reactions on both the heavy and light strands were performed at the Center for Genomic Technologies, the Hebrew University of Jerusalem, Israel, using DNA analyzer ABI 3500 (Applied Biosystems, USA).

Laboratory 3 (Moscow): DNA was extracted from 20–30 mg of bone powder from each of the samples from the Duvanny Yar using a phenol/chloroform protocol following overnight incubation with proteinase K at 37 °C. PCR amplification was performed using the same 18 primer pairs as for *U. glacialis* for 45 cycles as follows: 94 °C–30 sec, 50 °C–45 sec, 72 °C–45 sec. PCR products were analyzed using electrophoresis in 6% PAAG with subsequent staining with ethidium bromide and visualization in the UV light. PCR fragments were purified for sequencing by electrophoresis in 6% PAAG and Wizard columns (Promega, USA).

Laboratory 4 (Penza): Samples examined here were obtained (1) from museum collections and were represented by dry skin and teeth, and (2) from ethanol-preserved liver samples (Supplementary Table S1). Approximately 10–20 mg or 3–4 mm^3^ of tissue were sub-sampled from each specimen for DNA analysis. In order to minimize the damage of destructive sampling in museum specimens, ungual phalanges of the fourth finger of the forelimb were sampled and the skin was used to extract DNA. Tissue samples were homogenized by grinding in a 1.5-ml tube, incubated in 0.5 ml of STE buffer for 30 to 60 min, and then centrifuged. DNA was isolated from the pellet using a standard procedure that included overnight treatment with sodium dodecyl sulfate and proteinase K at 50 °C and subsequent phenol extraction. A 1316-bp mtDNA fragment covering the full-size *cyt b* gene flanked by the genes for tRNAs was amplified using primers specific for Family *Marmotinae*, designed from the sequenced mtDNA gene for tRNA of the three species (*Sciurus vulgaris, Glis glis* and *Spermophilus citellus*). PCR was carried out in a final volume of 25 μl, containing 50 mM Tris–HCl (pH 8.9); 20 mM ammonium sulphate, 20 μM EDTA; 150 μg/ml bovine serum albumin; dNTPs (200 μM of each); 2 mM MgCl_2_, 15 pmol of each primer (Supplementary Table S2); 2 units of *Taq* polymerase; and 0.1–0.2 μg DNA. Amplification was performed as follows: 94 °C for 1 min, 61 °C for 1 min, 72 °C for 3 min (30 cycles) for the pair of primers L-Glu-Sc/H-Pro-Sc. When using the internal primers (L-Glu-Sc/H830-Sfe and H830-Sfe/L397-Sp) the averaged reaction conditions were: 94 °C for 1 min; 59 °C for 1 min; and 72 °C for 2 min (for 30 cycles). PCR products were analyzed using electrophoresis in 6% PAAG with subsequent staining with ethidium bromide and visualization in the UV light. PCR fragments were purified for sequencing by electrophoresis in 6% PAAG and Wizard columns (Promega, USA).

Sequencing of the Duvanny Yar and museum samples was performed at the Genome Common Use Center at the Engelhardt Institute of Molecular Biology of the Russian Academy of Sciences using the BigDye Terminator v3.1 Cycle Sequencing Kit on ABI PRISM^®^ 3100-Avant Genetic Analyzer.

### Sequence alignments and diversity measures

The sequences were checked manually and validated using Sequencher 5.0 software (Genecodes, USA) for ambiguities and errors. Approved sequences were aligned using Geneious Pro 5.6.6 (BioMatters, New Zealand). We used DnaSP v.5.10 to calculate haplotype diversity (*h*) and mean interspecific *p*-distances for the *U. parryii* datasets^29^.

### Phylogenetic analyses

The entire dataset included 27 sequences obtained in the present study along with 45 previously published ones. Genbank accession numbers of the latter are as follows: Arc clade: 1–JF314404, 2–JF314409, 3–JF314414, 4–JF314416, 5–AY427983, 6–AY427988, 7–JF314423; Ber clade: 8–JF314428, 9–JF314430, 10–JF314431, 11–JF314432, 12–JF314436, 13–JF314441, 14–JF314446, 15–AY427998, 16–JF314451, 17–JF314456, 18–JF314461, 19–AY427980, 20–JF314466, 21–JF314471; SW clade: 22–HM204709, 23–AY427977, 24–GU220824, 25–AY428000, 26–GU220833, 27–GU220843, 28–GU220828, 29–AY427990; 30–GU220849, 31–GU220868, 32–GU220854, 33–AY427982, 34–AY427981, 35–GU220859; SE clade: 36–JF314478, 37–AY428018, 38–AY428012, 39–AY428008, 40–JF314487, 41–JF314492, 42–AY428017, 43–JF314500, 44–AY428015, 45–AY428009. *U. richardsonii* (JF314507) and *U. columbianus* (JF314509) were used as outgroup.

A median-joining network of 55 *cyt b* haplotypes was constructed using PopART v.1.7 software^30^. A maximum likelihood (ML) reconstruction of the phylogeny was performed using PhyML 3.0 software^39^ with 100 bootstrap runs, based on the complete *cyt b* sequence. The optimal model for the dataset (Hasegawa-Kishino-Yano model, Gamma distributed (HKY+G) was determined via jModelTest v.2.0^40^. The same dataset was examined using Bayesian Evolutionary Analysis by Sampling Trees (BEAST) v1.8.0 [ref. 41]. Here, the analysis was performed using the Hasegawa, Kishino & Yano nucleotide substitution model, Gamma distributed, with 4 categories under a strict molecular-clock model and a Bayesian skyline plot model, the latter allowing more flexibility of past population dynamics, and using a random starting tree model and normal priors. To attach a timescale to the phylogenetic estimate, we applied the radiocarbon ages of the ancient specimens as independent calibration information. For MCMC (Markov chain Monte Carlo) analysis, we used default settings in BEAUti (Bayseian Evolutionary Analysis Utility) that included a total sampling period of 30,000,000 steps, with samples drawn every 1,000 steps. The performance of the BEAST runs (convergence and ESS values superior to the critical threshold of 200) was tested using Tracer v1.5 [ref. 42]. The resulting trees were analyzed using TreeAnnotator (part of the BEAST v1.8.0 package) to compute a maximum clade probability tree with a burn-in of 10% and posterior probability limit of 0.5. The tree was viewed in FigTree v1.4. TipDatingBeast R package was used to assist the implementation of phylogenetic tip-dating tests using BEAST^30^.

## Additional Information

**Accession Codes:** Sequences generated in this study have been deposited in the GenBank (http://www.ncbi.nlm.nih.gov) with accession numbers KX646799-KX646825.

**How to cite this article**: Faerman, M. *et al*. DNA analysis of a 30,000-year-old *Urocitellus glacialis* from northeastern Siberia reveals phylogenetic relationships between ancient and present-day arctic ground squirrels. *Sci. Rep.* **7**, 42639; doi: 10.1038/srep42639 (2017).

**Publisher's note:** Springer Nature remains neutral with regard to jurisdictional claims in published maps and institutional affiliations.

## Supplementary Material

Supplementary Information

## Figures and Tables

**Figure 1:**
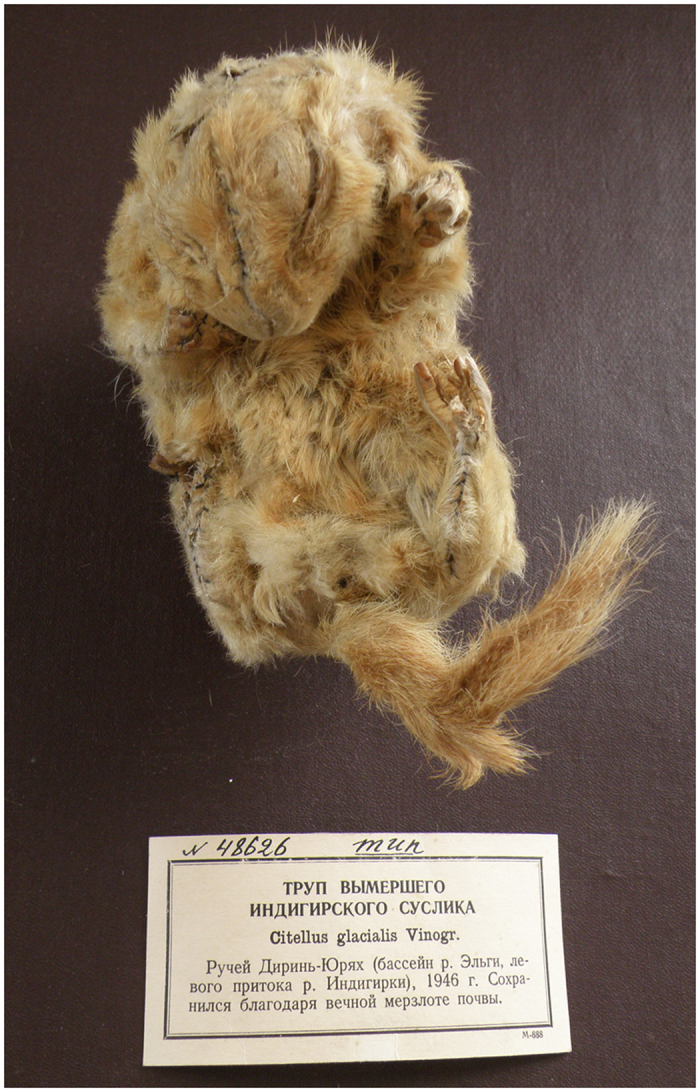
Mummified carcass of *U. glacialis* Vinogr., lectotype, ZIN-48626. ^14^C and DNA analyses were performed on *U. glacialis*, paralectotype, ZIN-34046. Both, the lectotype and the paralectotype, are currently stored at the Zoological Institute of the Russian Academy of Sciences, St. Petersburg, Russia (ZIN RAS). Reproduced with permission of ZIN RAS.

## References

[b1] Solzhenitsyn A. I. The Gulag Archipelago, 1918–1956: An Experiment in Literary Investigation (Harper & Row, 1974).

[b2] Popov Ju. N. Ten thousand years in permafrost. Mummies of fossil susliks. Sovetskaja Kolyma 79(3577), 4 Russian (1948).

[b3] Popov Ju. N. New findings of carcasses of Pleistocene animals in north-east USSR. Priroda 3, 75–76 Russian (1948).

[b4] Vinogradov B. S. On the discovery of carcasses of fossil arctic ground squirrels from the permafrost of the Indigirka river basin. Dokl. Akad. Nauk SSSR 62, 553–556 Russian (1948).

[b5] Gromov I. M., Bibikov D. I., Kalabukhov N. I. & Mejer M. N. Ground squirrels (*Marmotinae*). Fauna of USSR, mammals 3, 216–218 Russian (Nauka, 1965).

[b6] Helgen K. M., Cole F. R., Helgen L. E. & Wilson D. E. Generic revision in the Holarctic ground squirrel genus Spermophilus. J. Mammal. 90, 270–305 (2009).

[b7] Harrison R. G., Bogdanowicz S. M., Hoffmann R. S., Yensen E. & Sherman P. W. Phylogeny and evolutionary history of the ground squirrels (*Rodentia: Marmotinae*). J. Mammal. Evol. 10, 249–276 (2003).

[b8] Herron M. D., Castoe T. A. & Parkinson C. L. Sciurid phylogeny and the paraphyly of Holarctic ground squirrels (*Spermophilus*). Mol. Phylogenet. Evol. 31, 1015–1030 (2004). 15120398 10.1016/j.ympev.2003.09.015

[b9] Eddingsaas A. A., Jacobsen B. K., Lessa E. P. & Cook J. A. Evolutionary history of the arctic ground squirrel (*Spermophilus parryii*) in Nearctic Beringia. J. Mammal. 85, 601–610 (2004).

[b10] Cook J. A., Eddingsaas A. A., Loxterman J. L., Ebbert S. & MacDonald S. O. Insular arctic ground squirrels (*Spermophilus parryii*) of the North Pacific: indigenous or exotic? J. Mammal. 91, 1401–1412 (2010).

[b11] Galbreath K. E., Cook J. A., Eddingsaas A. A. & Dechaine E. G. Diversity and demography in Beringia: multilocus tests of paleodistribution models reveal the complex history of arctic ground squirrels. Evolution 65, 1879–1896 (2011). 21729045 10.1111/j.1558-5646.2011.01287.x

[b12] McLean B. S., Jackson D. J. & Cook J. A. Rapid divergence and gene flow at high latitudes shape the history of Holarctic ground squirrels (*Urocitellus*). Mol. Phyl. Evol. 102, 174–188 (2016). 10.1016/j.ympev.2016.05.040 27261251

[b13] Hofreiter M. & Stewart J. Ecological change, range fluctuations and population dynamics during Pleistocene. Curr. Biol. 19, R584–R594 (2009). 19640497 10.1016/j.cub.2009.06.030

[b14] Lorenzen E. D. . Species-specific responses of Late Quaternary megafauna to climate and humans. Nature 479, 359–364 (2011). 22048313 10.1038/nature10574 PMC4070744

[b15] Prost S. . Losing ground: past history and future fate of Arctic small mammals in a changing climate. Glob. Chang. Biol. 19, 1854–1864 (2013). 23505210 10.1111/gcb.12157

[b16] Shapiro B. . Rise and fall of the Beringian steppe bison. Science 306, 1561–1565 (2004). 15567864 10.1126/science.1101074

[b17] Stiller M. . Withering away-25,000 years of genetic decline preceded cave bear extinction. Mol. Biol. Evol. 27, 975–978 (2010). 20335279 10.1093/molbev/msq083

[b18] Palkopoulou E. . Complete genomes reveal signatures of demographic and genetic declines in the woolly mammoth. Curr. Biol. 25, 1395–1400 (2015). 25913407 10.1016/j.cub.2015.04.007 PMC4439331

[b19] Orlando L. . Recalibrating *Equus* evolution using the genome sequence of an early Middle Pleistocene horse. Nature 499, 74–78 (2013). 23803765 10.1038/nature12323

[b20] Ersmark E. . Population demography and genetic diversity in the Pleistocene Cave Lion. Open Quaternary 1, 1–15, http://dx.doi.org/10.5334/oq.aa (2015).

[b21] Lee E. J. . Ancient DNA analysis of the oldest Canid species from the Siberian Arctic and genetic contribution to the domestic dog. PLoS ONE 10, e0125759, doi: 10.1371/journal.pone.0125759 (2015). 26018528 PMC4446326

[b22] Harington C. R. Annotated Bibliography of Quaternary Vertebrates of Northern North America—with Radiocarbon Dates (University of Toronto Press, 2003).

[b23] Zazula G. D., Froese D. G., Elias S. A., Kuzmina S. & Mathewes R. W. Arctic ground squirrels of the mammoth-steppe: paleoecology of late Pleistocene middens (similar to 24000-29450 c-14 yr BP), Yukon Territory, Canada. Quat. Sci. Rev. 26, 979–1003 (2007).

[b24] Zazula G. D., Froese D. G., Elias S. A., Kuzmina S. & Mathewes R. W. Early Wisconsinan (MIS 4) arctic ground squirrel middens and a squirrel-eye-view of the mammoth-steppe. Quat. Sci. Rev. 30, 2220–2237 (2011).

[b25] Boeskorov G. G. & Beloljubsky I. N. A fossil suslik from the lower Kolyma river. Otechestvennaja geologija 5, 38–40 Russian (2000).

[b26] Zanina O. G., Gubin S. V., Kuzmina S. A., Maximovich S. V. & Lopatina D. A. Late-Pleistocene (MIS 3–2) palaeoenvironments as recorded by sediments, palaeosols, and ground-squirrel nests at Duvanny Yar, Kolyma lowland, northeast Siberia. Quat. Sci. Rev. 30, 2107–2123 (2011).

[b27] Boom R. . Rapid and simple method for purification of nucleic acids. J. Clin. Microevol. 28, 495–503 (1990). 10.1128/jcm.28.3.495-503.1990 PMC269651 1691208

[b28] Höss M. & Pääbo S. DNA extraction from Pleistocene bones by silica-based purification method. Nucleic Acids Res. 21, 3913–3914 (1993). 8396242 10.1093/nar/21.16.3913 PMC309938

[b29] Librado P. & Rozas J. DnaSP v5: A software for comprehensive analysis of DNA polymorphism data. Bioinformatics, 25 1451–1452 (2009). 19346325 10.1093/bioinformatics/btp187

[b30] Leigh J. W. & Bryant D. POPART: full-feature software for haplotype network construction. Methods Ecol. Evol. 6, 1110–1116 (2015).

[b31] Rieux A. & Khatchikian C. E. TipDatingBeast: an R package to assist the implementation of phylogenetic tip-dating tests using BEAST. Mol. Ecol. Resour. Accepted Author Manuscript, doi: 10.1111/1755-0998.12603 (2016). 27717245

[b32] Elias S. A. & Brigham-Grette J. Late Pleistocene glacial events in Beringia Encyclopedia of Quaternary Science, 2nd ed. (ed. Scott E.) 191–201 (Elsevier Science Publishers B.V.; North-Holland, 2013).

[b33] Arkhipov S. A., Isaeva L. L., Beslay V. G. & Glushkova O. Glaciation in Siberia and north-east USSR. Quat. Sci. Rev. 5, 463–474 (1986).

[b34] Clark P. U. . The Last Glacial Maximum. Science 325, 710–714 (2009). 19661421 10.1126/science.1172873

[b35] Boaretto E. . Radiocarbon dating of charcoal and bone collagen associated with the Early Pottery at Yuchanyan Cave, Hunan Province, China. Proc. Natl. Acad. Sci. USA 106, 9595–9600 (2009). 19487667 10.1073/pnas.0900539106 PMC2689310

[b36] Reimer P. J. . IntCal13 and Marine13 radiocarbon age calibration curves 0–50,000 years cal BP. Radiocarbon 55, 1869–87 (2013).

[b37] Bronk Ramsey C. Radiocarbon calibration and analysis of stratigraphy: The OxCal program. Radiocarbon 37, 425–430 (1995).

[b38] Bronk Ramsey C. Development of the radiocarbon calibration program OxCal. Radiocarbon 43, 355–363 (2001).

[b39] Guindon S. . New algorithms and methods to estimate maximum-likelihood phylogenies: assessing the performance of PhyML 3.0. Syst. Biol. 59(3), 307–321 (2010). 20525638 10.1093/sysbio/syq010

[b40] Darriba D., Taboada G. L., Doallo R. & Posada D. jModelTest 2: more models, new heuristics and parallel computing. Nat. Methods 9(8), 772–772 (2012). 10.1038/nmeth.2109 PMC4594756 22847109

[b41] Drummond A. J., Suchard M. A., Xie D. & Rambaut A. Bayesian phylogenetics with BEAUti and the BEAST 1.7. Mol. Biol. Evol. 29, 1969–1973 (2012). 22367748 10.1093/molbev/mss075 PMC3408070

[b42] Rambaut A. & Drummond A. J. Tracer, v.1.5. http://beast.bio.ed.ac.uk/Tracer (2007).

