# Supplementary material for: DNA analysis of a 30,000-year-old Urocitellus glacialis from northeastern Siberia reveals phylogenetic relationships between ancient and present-day arctic ground squirrels
Source: Sci Rep. 2017 Feb 16;7:42639. doi: 10.1038/srep42639 (PMC5311991; doi:10.1038/srep42639)
Supplement: Supplementary Information [file srep42639-s1.pdf]

Title

DNA analysis of a 30,000-year-old *Urocitellus glacialis* from northeastern Siberia reveals phylogenetic relationships between ancient and present-day arctic ground squirrels

Authors

Marina Faerman[1]*, Gila K. Bar-Gal[2], Elisabetta Boaretto[3], Gennady G. Boeskorov[4], Nikolai E. Dokuchaev[5], Oleg A. Ermakov[6], Fedor N. Golenishchev[7], Stanislav V. Gubin[8], Eugenia Mintz[3], Evgeniy Simonov[9-11], Vadim L. Surin[12], Sergei V. Titov[6], Oksana G. Zanina[8], Nikolai A. Formozov[13]*

[1]Laboratory of Bioanthropology and Ancient DNA, Faculty of Dental Medicine, The Hebrew University of Jerusalem, Jerusalem 91120, Israel

[2]Koret School of Veterinary Medicine, The Robert H. Smith Faculty of Agriculture, Food & Environment, The Hebrew University of Jerusalem, Rehovot 76100, Israel

[3]D-REAMS Radiocarbon Laboratory, Scientific Archaeology Unit, Weizmann Institute of Science, Rehovot 76100, Israel

[4]Diamond and Precious Metals Geology Institute of the Siberian Branch of the Russian Academy of Sciences, Yakutsk 677007, Russian Federation

[5]Institute of Biological Problems of the North, Far-East Branch of the Russian Academy of Sciences, Magadan 685000, Russian Federation

[6]Department of Zoology and Ecology, Penza State University, Penza 440026, Russian Federation

[7]Laboratory of Theriology, Zoological Institute, Russian Academy of Sciences, Saint Petersburg 199034, Russian Federation

[8]Soil Cryology Laboratory, Institute of Physicochemical and Biological Problems in Soil Science, Russian Academy of Sciences, Pushchino 142290, Russian Federation

[9]Papanin Institute for Biology of Inland Water, Russian Academy of Sciences, Borok 152742, Russian Federation

[10]Institute of Systematics and Ecology of Animals, Siberian Branch of Russian Academy of Sciences, Novosibirsk 630091, Russian Federation

[11]Tomsk State University, Tomsk 634050, Russian Federation

[12]National Research Center for Hematology, Russian Ministry of Health, Moscow 125167, Russian Federation

[13]Department of Vertebral Zoology, Faculty of Biology, Lomonosov Moscow State University, Moscow 119991, Russian Federation

Supplementary Information

Radiocarbon dating

A liver sample of *Urocitellus glacialis* (Gla1) was analyzed for radiocarbon dating. In the table below the details of the sample are given, including the ^{14}C age and the calibrated ranges in years BP (before present). The calibrated ranges are given for ±1σ (68.2% probability), and for ±2σ (95.4% probability).

RTK	Sample ID	C %	$\delta^{13}C$ ‰ PDB	^{14}C age±1σ years BP	Calibrated age±1σ, years BP	Calibrated age±2σ, years BP
6386	ZIN-34046	48	-21.4	29,045 ± 925	33,990- 31,990 (68.2%)	34,920- 31,250 (95.4%)

^{14}C age is reported in conventional radiocarbon years (before present =1950) in accordance with international convention[43]. Calibrated ages in calendar years have been obtained from the calibration tables[36] by means of the 2010 version OxCal v. 4.2.4 of Bronk-Ramsey[37,38]. The probability distribution of the calibrated ranges is given in Figure 3.
Preservation products were reported to have been applied to the sample from its discovery in 1946[3,4]. Since there is no secure way to eliminate the chemical preservatives applied (in this case, ethanol and possibly but not necessarily formalin) we used Fourier Transform Infrared Analysis (FTIR) to determine the presence of formaldehyde in the liver sample, before and after pre-treatment for collagen extraction following a procedure based on the acid- alkali- acid (AAA) technique and described elsewhere[35]. The FTIR spectrum showed only the presence of the Amide I, II and hydroxiproline (1640, 1550, 1450 cm^{-1} respectively). No peaks related to the formaldehyde were detected.

43. Stuiver, M. & Polach, H.A. *Radiocarbon* **19**, 355 (1977)

Table S1: List of examined ancient and modern arctic ground squirrels

Locality number	Locality name	Latitude and longitude	Voucher code	Collector, Date	Tissue sampled	Sequence name	*Cyt b* GenBank accession number
1	El'ga R., Yakutia	64.37°N 142.00°E	ZIN-34046	Popov Ju.I., 1946	Bone Skin Liver	Gla 1	KX646799
2	Duvanny Yar, NE Yakutia	68.63°N 159.11°E	IGDPM - 6391	Beloljubsky I.N., 1987	Bone	Gla 2a	KX646800
			P-1311	Zanina O.G., 2002	Bone	Gla 2b	KX646801
			P-1320	Zanina O.G., 2002	Bone	Gla 2c	KX646802
			P-Up4	Zanina O.G., 2002	Bone	Gla 2d	KX646803
3	Verkhoyansk, Yakutia	67.61°N 133.30°E	PSU-510	2005, NA	Liver	BerR 3	KX646804
4	Tuostakh R., Yakutia	67.85°N 136.37°E	S-160600	Emel'yanova L.G., 1988	Skin	BerR 4	KX646805
5	Ust'-Nera, Yakutia	63.74°N 146.10°E 63.74°N 146.10°E	IBPN-5563 IBPN-5566	Berman D.I., 1978	Tooth Tooth	BerR 5a BerR 5b	KX646806 KX646807
6	Kolyma R.,	61.86°N	NED-29	Dokuchaev	Skin	BerR 6	KX646808

	Magadanskaya oblast	147.61°E		N.E., 2010			
7	Beryezovka R., Yakutia	67.59°N 155.53°E	IGDPM, no number	1964, NA, passed by Boeskorov G.G.	Skin	BerR 7	KX646809
8	Omolon R., Chukotka	66.07°N 159.17°E	IBPN-2425	Korolenko G.E., 1972	Skin	BerR 8	KX646810
9	Malyj Anyuy R., Chukotka	67.39°N 168.35°E	IBPN-6152	Kiryushchenko S.P., 1972	Skin	BerR 9	KX646811
10	Ust'-Chaun, Chukotka	68.78°N 170.49°E	S-88325	Krivosheev V.G., 1969	Skin	BerR 10	KX646812
11	Anadyr' R. (upper), Chukotka	66.46°N 169.34°E	NED-304	Dorogoy I.V., 2003	Skin	BerR 11	KX646813
12	Penzhina R., Koryak okrug	62.44°N 166.20°E	S-176182	Dolgov V.A., 1985	Skin	BerR 12	KX646814
13	Glubokaya Bay, Koryak okrug	61.09°N 172.10°E	S-46688	Sleptsov M., 1947	Skin	BerR 13	KX646815
14	Guba Khychak, Chukotka	69.81°N 173.71°E	IBPN-389	Krivosheev V.G., 1970	Skin	BerR 14	KX646816
15	Kanchalan-2 R. (middle), Chukotka	65.54°N 177.24°E	IBPN-4113	Chernyavsky F.B., 1975	Skin	BerR 15	KX646817
16	Kanchalan-1 R. (upper),	66.17°N 179.25°E	IBPN-4112	Chernyavsky F.B., 1975	Skin	BerR 16	KX646818

	Chukotka						
17	Kolyuchinskaya Guba, Chukotka	67.04°N 174.58°W	S-138816	Tomkovich P.S., 1986	Skin	BerR 17	KX646819
18	Arakamchechen Island, the Bering Sea	64.76°N 172.40°W	S-129779	Dolgov V.A., 1982	Skin	BerR 18	KX646820
19	Uelen, Chukotka	66.15°N 169.76°W	S-112267	Tomkovich P.S., 1978	Skin	BerR 19	KX646821
20	Avacha, Kamchatka	53.17°N 158.75°E	ZIN-67213	Expedition ICG, NA	Skin	Kam 20	KX646822
21	Kronotskaya R., Kamchatka	54.66°N 160.45°E	PSU-2/2011	Shpilenok I., 2011*	Liver	Kam 21	KX646823
22	Ust'-Kamchatsk, Kamchatka	56.25°N 162.50°E	PSU-6/2011	Zhitkov B.M., 1974, expedition IGE	Skin	Kam 22	KX646824
23	Tigil', Kamchatka	57.76°N 158.67°E	ZIN-67204	expedition ICG, NA	Skin	Kam 23	KX646825

Locality numbers cross-reference Figure 2. Sequence names cross-reference Figures 4 and 5.

ZIN - Zoological Institute of the Russian Academy of Sciences (St. Petersburg, Russian Federation); IGDPM - Diamond and Precious Metals Geology Institute, Siberian Branch of the Russian Academy of Sciences (Yakutsk, Russian Federation); P - Institute of Physicochemical and Biological Problems in Soil Science of the Russian Academy of Sciences (Pushchino, Russian Federation); PSU - Penza State University (Penza, Russian Federation); S - Zoological Museum of the Moscow State University (Moscow, Russian

Federation); IBPN - Institute of Biological Problems of the North, Far-East Branch of the Russian Academy of Sciences (Magadan, Russian Federation); IGE - Russian Research Institute of Game Management and Fur Farming, Russian Academy of Sciences (Kirov, Russian Federation), ICG - Institute of Cytology and Genetics, Siberian Branch of the Russian Academy of Sciences (Novosibirsk, Russian Federation); NED - Collection of N.E. Dokuchaev; NA - unknown. *We are very grateful to Alisa, the fox who ‘collected’ the first specimens from Kamchatka for this study and ‘presented’ them to Igor Shpilenok.

Table S2: List of primers and amplicon size in ancient (A), museum (B) and ethanol-preserved (C) samples

A. *Urocitellus parryii glacialis* and other fossil samples

No	Primer name and sequence	Nucleotide position in *Cyt b* gene	Amplicon size, bp
1	GlCbSTD AATGACATGAAAAATCATCGTTGT<br>GlCbSTR GCAGGTAAGTCGATAAAGGAGT	tRNA-Glu<br>68-47	113
2	GlCbD1 ATGACAAACATCCGCAAAACTC<br>GlCbR1a CTAGAAGAGACCCAAAGTTTCA	1-22<br>112-91	112
3	GlCbD2a ACCTCCAACATTTCTGCATGA<br>GlCbR2 CTGATGAAAAGGCTGTTATAGT	69-90<br>196-175	128
4	GlCbD3 ACTAGCAATACATTACACATCTGA<br>GlCbR3a AATATAGATGCGCCGTTAGCAT	151-173<br>269-248	119
5	GlCbD4a TGGTTGACTAATCCGCTATATAC<br>GlCbR4a AGAATGACTCCAATGTTTCATGT	225-247<br>356-334	132
6	GlCbD5 CTATGGCTCATATACTTACTTTGA<br>GlCbR5a GGTTAGTAATTACAGTTGCTCC	309-332<br>445-424	115
7	GlCbD6 CTGAGGTCAAATGTCATTCTGA<br>GlCbR6 GAGTAGCTTTAATCTACTGAGAA	402-423<br>523-502	122
8	GLCbD7 TAGTAGAATGAATTTGAGGTGG<br>GlCbR7 TGAAGGAAAAGAAGGTGAACTAT	479-500<br>602-580	124
9	GlCbD8a CCATTTATTATCGCAGCTCTA<br>GlCbR8a GATATCTTTGATGGTGTAATATGG	656-676<br>687-664	132
10	GlCbD9 GATTCAGATAAAGTCCCCTTTCA<br>GlCbR9 CTATAATTATCAGGGTCTCCTAG	640-662<br>770-748	131
11	GlCbD10a ACTCTAGTCCTATTTTCACCTGA<br>GlCbR10 TTGTTGGGGATAGATCGGAG	721-743<br>860-841	140
12	GlCbD11 TTCCTATTTGCCTACGCTATC<br>GlCbR11 GCTTCGTTGTTTAGATAGATG	820-840<br>942-922	123
13	GlCbD12a TCAATTCTCATCCTAATACTTTTCC<br>GlCbR12a ATTCAGGTTAACGTAAATAGGTC	889-913<br>1013-991	125
14	GlCbD13a GCATATTCTGAATTCTAGTAGCA<br>GlCbR13ax AATAGTGAAATATAGGATTGATGC	968-990<br>1083-1060	116
15	GlCbD14a CCCATATATTATTATCGGCCAAC<br>GlCbR13 TCTTCATTTAAGAAGTTTGTTTTC	1035-1057<br>1140-1017	106
16	GlCbendD CACTATTATTCTCCTAATCTTACC<br>GlCbendR CTTCATTTTTGGTTTACAAGACCA	1077-1100<br>tRNA-Thr	114
17	GlCytD1 GATCTTCTAGGAGACCCTGA<br>GlCytR1 GATCGGAGGATAGCGTAGGC	742-761<br>848-829	107
18	GlCytD2 GCCTACGCTATCCTCCGATC<br>GlCytR2 GATGAAGTAGTGGGAAAAGTAT	829-848<br>925-904	96

B. Museum dry specimens of *Urocitellus parryii*

No	Primer name and sequence	Nucleotide position in *Cyt* b gene	Amplicon size, bp
19	GlCbSTD AATGACATGAAAAATCATCGTTGT<br>CBPR1 CTCCAATGTTTCATGTTTCAAAGT	tRNA-Glu<br>349-326	394
20	CBPD2 CTCTTTCTTCATGTAGGCCGA<br>CBPR2 GTTTCGTGAAGGAAAAGAAGGT	280-300<br>608-587	329
21	CBPD3 TTCTTCCATTTATTATCGCAGC<br>CBPR3 GTTTGTTGGGGATAGATCGGA	551-572<br>862-842	312
22	CBPD4 TTCCTATTTGCCTACGCTAT<br>GlCbendR CTTCATTTTTGGTTTACAAGACCA	820-839<br>tRNA-Thr	421

C. Ethanol-preserved specimens of *Urocitellus parryii**

No	Primer name and sequence	Nucleotide position in *Cyt* b gene	Amplicon size, bp
23	Glu-Sc AACCATGACCAATGACATGAAAAATCA<br>Pro Sc GAATATCAGCTTTGGGAGTTGAAGGTGGA	tRNA-Glu<br>tRNA-Pro	1316
24	Glu-Sc AACCATGACCAATGACATGAAAAATCA<br>830-Sfe GGRATAGATCGGAGAATAGCGTAGGC	tRNA-Glu<br>854-829	908
25	397-Sp CTTCCCTGAGGCCAAATATCATT<br>Pro Sc GAATATCAGCTTTGGGAGTTGAAGGTGGA	tRNA-Pro<br>397-419	866

* These primers were designed by V.S. Lebedev.

Table S3: Polymorphic sites observed in complete *cytochrome b* gene

```
                                                                               11111111111111
             11111112222222223333333333444445556666666666677777778888899999999900000000011111
          67800234660023466780012255567467781470222355567711226890146800122777804445788900012
          02928454254784947064623812408821293630046424794624178621907504417059020350406614668
CONS      ctgatgcattctcattgtctacttcacgatgagatcttaccggccccagcaatacctcggtactagtatattatccttaccct
Gla1      .c...at..c..t...a.a..tc...t.g...a......t.a.t.t..a..gc...c..ac.tc.ac..ga..ctt..g.t.c
Gla2a     .c...at..c..t...a....tc...t.g...a......t.a.t.t....ggc......ac..c.ac..ga..c.t..g.t.c
Gla2b     .c...at..c.ctt..a...ctc...t.g...a......t.a.t.t..a..gc...c..ac..c.ac..ga..c.t..g.t.c
Gla2c     .c...at..c..t...a....tc...t.g...a....c.t.a.t.t..a..gc...c..ac..c.ac..ga..c.t..g.t.c
Gla2d     .c...at..c..t...a....tc...t.g...a......t.a.t.t..a..gc...c..ac..c.ac..ga..c.t..g.t.c
Kam21     tc...at..c..t...a....tcct.t.gc.tag.....t.a.t.t.g...gc...c.aac..cgacg.ga..c.t..g.ttc
Kam20     tct..at..c..t...a....tc.t.t.gc.tag.....t.a.t.t.g...gc...c.aac..cgacgcga..c.t..g.ttc
Kam23     tct..at..c..t...a....tc.t.t.g..tag.....t.a.t.t.g...gc...ctaac..cgacg.ga..c.t..g.ttc
Kam22     tct..at..c..t...a....tc.t.t.g..tag....tt.a.t.t.g...gc...ctaac..cgacg.ga..c.t..g.ttc
BerR17    .c.gc....................g.a.....................t.....tc..........................
BerR13    ...gc.....t................a.....................t......c..........................
BerR14    ...gc.....t................a..............a......t......c..........................
BerR4     ...............a.c...........ca.....c..................tct...g..............c......
BerR3     .................c...........ca.....c...................c...................c......
BerR15    ...g...............t....................t.............t..t.............c....c..t...
BerR19    ...g....c............................................gt.....................c......
BerR18    ...g....c.............................................t..t..................c......
BerR5b    ...g...............t....................t.............t.................g...c......
BerR8     ...g...............t....................t.............t..t..............g...cc.....
BerR16    ...g...............t....................t.............t..t..............g...c......
BerR12    ...g...g......c....t..............ct....t.....t.......t.................g...c......
BerR5a    .........................t.........................................................
BerR7     .........................t.........................................................
BerR6     .........................t.........................................................
```

CONS - consensus *cytochrome b* gene sequence of modern *U. parryii* from northeastern Siberia. Specimen names cross-reference Supplementary Table S1. Unique polymorphic sites of the *glacialis* lineage are marked in red.

Table S4: Mean and 95% HPD estimates of TMRCA of the main nodes based on BEAST analysis calibrated with the radiocarbon ages of the ancient specimens

Node number	Node description	Mean, years BP	95% HPD	Posterior probabilities
1	*U.parryii/U. richardsoni* and *U. columbianus*	278,521	143,266-447,893	1
2	Gla/Kam/SW/SE/Ber/BerR and Arc/*U. richardsoni*	122,662	66,919-192,091	1
3	Gla/Kam/SW and SE/Ber/BerR	104,434	58,990-161,779	0.92
4	Arc and *U. richardsoni*	84,832	37,665-142,412	0.99
5	SW and Gla/Kam	65,490	42,216-96.117	1
6	Gla and Kam	48,960	37,029-65,285	1
7	SW	31,937	15,322-51,354	1
8	SE and Ber/BerR	43,886	21,595-71,605	1
9	SE	18,364	7,080-33,146	1
10	Ber/BerR	28,926	13,880-46,901	1
11	subclade within Ber/BerR	20,425	8,738-34,486	0.98
12	subclade within Ber/BerR	24,711	12,048-40,484	0.89

Figure S1: Comparison of the substitution rates estimated using BEAST from both the original and 20 date-randomized datasets from the DRT (date-randomization test)

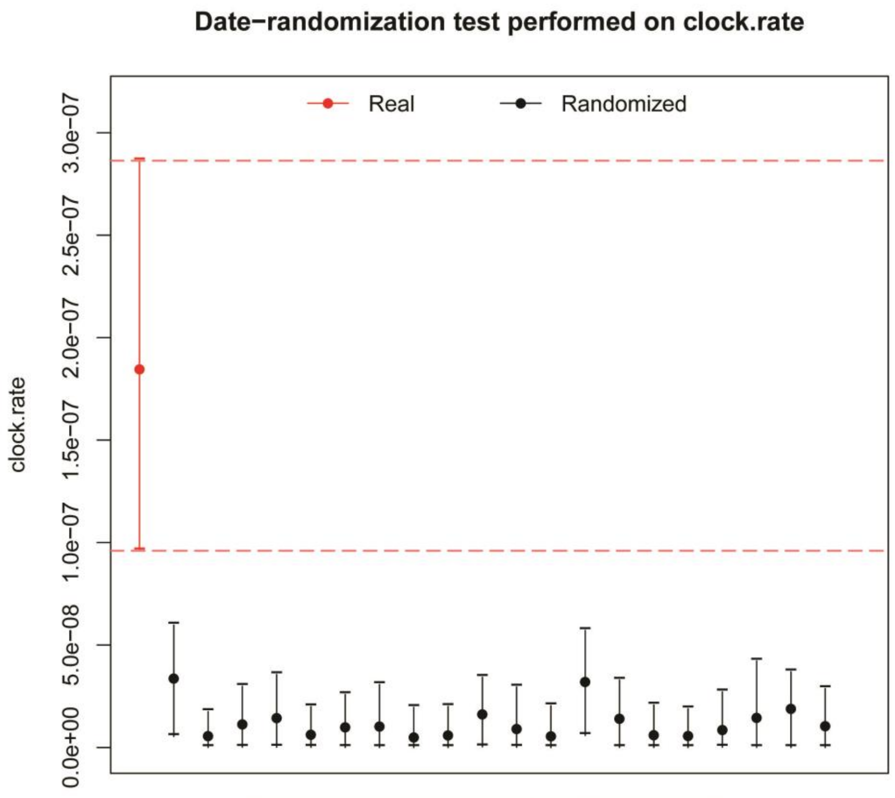

Date-randomization test (DRT) was applied to validate presence of temporal signal in the original dataset. The R package 'TipDatingBeast' [ref. 31] was used to assist DRT by generation of input files and analysis of BEAST output files. Molecular clock rate was estimated in original dataset along with 20 date-randomized datasets where calibration information (age of the specimens) were randomly placed among all tips. The mean clock rates with 95% HPD intervals were plotted and examined for possible overlaps. The examined dataset successfully passed the DRT showing no overlap with date-randomized datasets thus proving its utility for tip-dating analysis in BEAST.
